# Multiscale Modeling Is Required for the Patent Ductus Arteriosus in Preterm Infants

**DOI:** 10.3389/fped.2022.857434

**Published:** 2022-03-23

**Authors:** Harvey Ho, Xiaojuan Ji

**Affiliations:** ^1^Auckland Bioengineering Institute, The University of Auckland, Auckland, New Zealand; ^2^Department of Ultrasound, Chongqing General Hospital, University of Chinese Academy of Sciences, Chongqing, China

**Keywords:** ductus arteriosus, fetus, neonates, *in silico* modeling, circulation, hemodynamics

## Introduction

The ductus arteriosus (DA) in the fetus diverts most of the deoxygenated blood returning from the head, upper extremities and coronary sinus into the descending aorta, bypassing the pulmonary circulation (1, 2). The DA in full-term newborns closes within 24 to 48 h after delivery to facilitate the blood perfusion of lung tissues. However, DA often fails to close in preterm neonates. Indeed, 70% of preterm infants delivered before 28 weeks of gestation require surgical closure or pharmaceutical treatment (1). Patent DA (PDA) in preterm infants causes severe left-to-right shunting. The overloaded pulmonary perfusion causes pulmonary edema, bronchopulmonary dysplasia, pulmonary hypertension and other conditions such as hyperactive precordium and cardiomegaly (3, 4). From a hemodynamic perspective, the high resistance in the pulmonary bed and higher pressure in the pulmonary artery drive the blood flow through the DA into systemic circulation during fetal life. After delivery, the pressure gradient between pulmonary and systemic circulations reverses and exposes the pulmonary microvasculature to systemic blood pressure and increased pulmonary blood flow from PDAs (1, 5).

As a conduit for blood flow, it is critical to determine the so-called “hemodynamically significant” PDA (hsPDA), defined as the shunting of left-to-right blood flow that increases the pulmonary blood flow and decreases the systemic blood due to the amount of blood running from the descending aorta into the lung circulation (4), and imposes a threat to the survival of a preterm infant (6). Multi-center clinical trials, e.g., that described in (6), and animal (sheep) models (7) have investigated the hemodynamic implication of PDAs. The blood flow in DAs depends upon the pressure gradient between the pulmonary and systemic circulations. In the sheep model, reversed flow through the DA contributed up to 50% of total pulmonary blood flow at 30 min after the onset of pulmonary ventilation (7).

Underlying the hemodynamic phenomena in PDA and the pulmonary and systemic circulations are the fundamental changes in PDA's walls. In term infants, the smooth muscle layer of DA develops ischemic hypoxia, which drives the cell death and inflammatory cascade, leading to the remodeling of DA, and transforming the DA into a non-contractile ligament (8). In preterm infants, there is no vulnerable region of the wall that is at risk for loss of vasa vasorum flow, therefore the PDA is less likely to develop the severe degree of hypoxia that is necessary for ductus remodeling (8).

## Hemodynamics in the PDA Examined From Echocardiography

Echocardiography is the gold standard for the diagnosis of PDA (4, 5). Echocardiographic assessments include measuring the ductal size, ductal flow patterns, peak systolic and end-diastolic flow velocities, and the presence of retrograde diastolic flow (5, 9). The cut-off value for hsPDA is the ductal diameter ≥ 1.5 mm (4), which should be measured from 2D ultrasonic imaging rather than color Doppler as the latter may overestimate the ductal diameter (5). Besides the ductal diameter, the flow direction and pulsatile flow pattern are also valuable for assessing psPDA. Specifically, a pulsatile, non-restrictive left-to-right Doppler pattern is most sensitive (93.5%) and specific (100%) for predicting its development (5). Furthermore, several other parameters, such as the left ventricular output, volume and pressure, can be used as a surrogate for the degree of shunting at the ductal level (5). In addition, parameters for systemic hypo-perfusion e.g., the absent or retrograde diastolic flow in the abdominal aorta (4), may also be measured from echocardiography (5).

**Figure 1:**
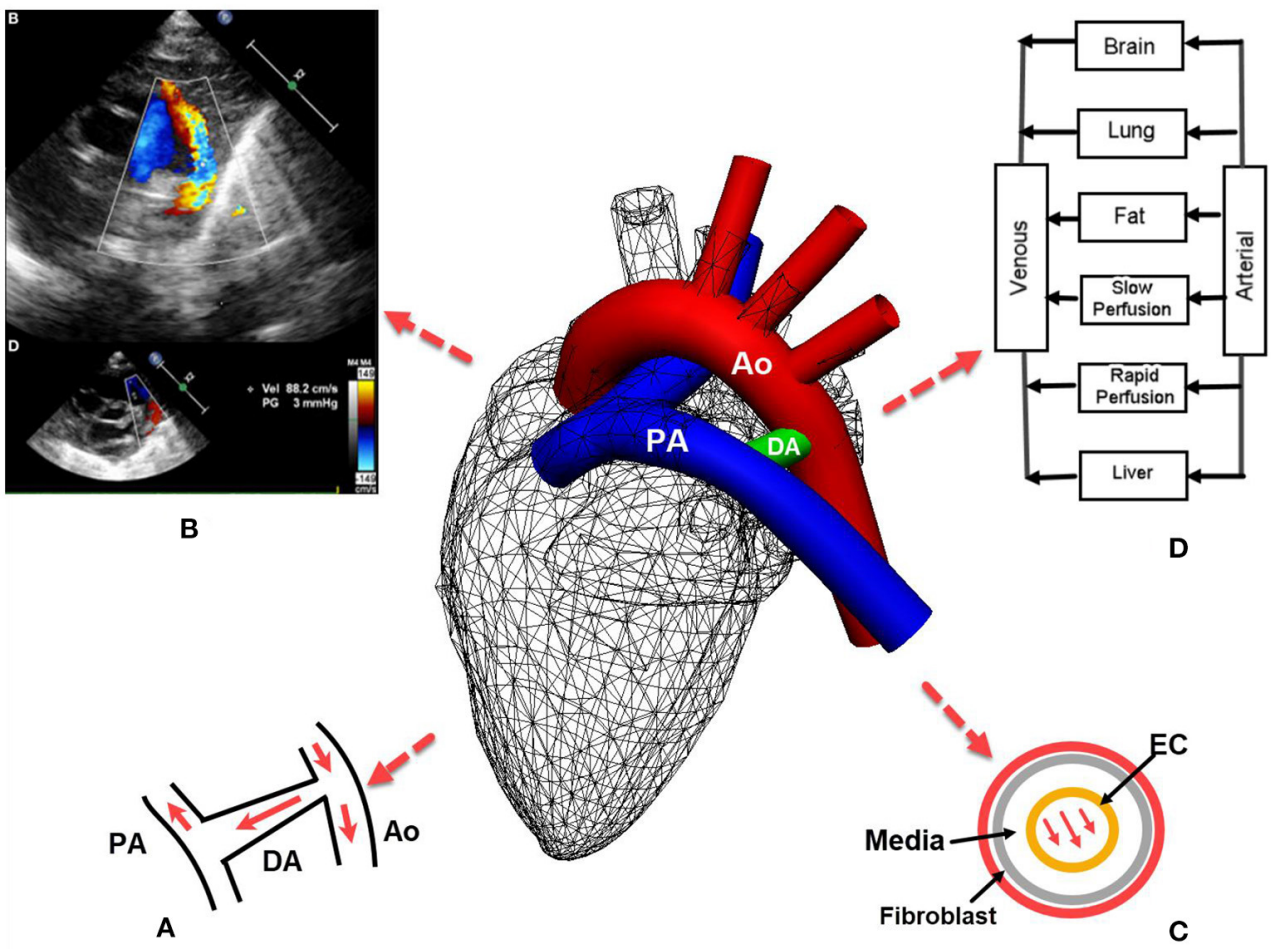
Patent ductus arteriosus (PDA) in preterm infants: **(A)** the left-to-right or reversed flow in PDA after birth. Note the constriction where the DA joins aorta introduces high pressure gradient according to the Bernoulli's equation; **(B)** echocardiography of PDA also reveals the pressure gradient between systemic and pulmonary circulations that aids hemodynamic modeling (4); **(C)** *in silico* models may be developed for the remodeling of different layers of the DA; **(D)** a typical pharmacokinetics model where blood flow rates are used to derive the drug amount in an organ. PA, pulmonary artery; Ao, Aorta; EC, endothelial cell.

Pertinent to hemodynamic modeling of PDAs, contemporary echocardiography can reveal the pressure gradient between the systemic and pulmonary circulations (Figure 1B) (4), which is of great value for providing boundary conditions for flow equations.

## Modeling for the Hemodynamics in PDA

*In silico* models have been used to simulate multiple aspects of the cardiovascular system. However, few have been developed for the circulation transitions from fetuses to neonates. Even less is the *in silico* models developed for PDAs. The PDA model in (10) uses two semi-cylinders (representing the pulmonary artery and aorta) joined by a plate (representing the DA), which is highly simplified and idealistic in its geometry. For *in silico* models to contribute to unraveling the key mechanisms underlying PDA, thoughts must be placed on the intimate connections between remodeling of the DA, the stimuli for vessel tone regulation, and the hemodynamics in it. Toward that end, we suggest a paradigm i.e., multiscale *in silico* modeling that closely links the hemodynamics in PDA with vessel constituents and vascular tones.

Hemodynamic models have been developed for the cardiovascular system of fetuses. For example, van den Wijingaard et al. designed a distributed model that consists of 13 arterial segments and nine vascular beds for the abnormal arterial flows in fetuses (11). Myers and Capper used a transmission line model to simulate the pulsatile arterial flow in the thoracic aorta and other arteries in fetuses (12). However, neither study included the DA and pulmonary artery in the arterial model. Blood flow simulations have been performed at a single-vessel level for the umbilical artery (13) and the ductus venosus (14). In a fetal circulation model, the DA is designed as an electronic component connecting the descending aorta and the pulmonary artery (15). Similar treatment is used where the model for PDA is based on PHYSBE and is modified in a way to include an additional flow derived from the aorta toward lungs (16).

Concerning modeling techniques, the *in silico* blood flow models mentioned above range from lumped parameter (or 0D) models to the transmission line and 1D models where the vessel geometry (length and diameter) is incorporated. If the purpose of the simulation is to reproduce the velocity waveform observed in echocardiography and the impedance of the vascular bed, then a coupled 1D and lumped parameter model, e.g., of (17), or coupled 1D and transmission line model, e.g., of (18) could serve the purpose. On the other hand, if the modeling goal is to investigate the detailed 3D flow patterns, e.g., the formation of vortex and flow separations after constriction in PDA, and the wall shear stress, then a 3D flow model should be applied (19, 20). A recent model simulated the 3D flow in the PDA to investigate the effects of the PDA on the flow features of the modified Blalock-Taussig shunt (21). Another study investigated the 3D flow in the PDA with respect to three typical pulmonary artery (PA) dysplasia structures and different sizes of PDA (22). Similarly, Hsia et al. developed a multi-dimensional model for the Norwood operation with a right ventricle-to-pulmonary artery shunt (23).

Hence, various modeling methods can be adopted for the left-to-right flow in the PDA in preterm infants, depending on the level of details required for the hemodynamics in the PDA. Szpinda et al. found that the reversed flow in the PDA was less than that expected from the pressure gradient (2). This is because the constriction at the joint between the PDA and the aorta introduces a higher pressure gradient according to the Bernoulli's equation (Figure 1A). Thus, the rapid flow at the constriction suits a flow model of a higher dimension (3D or 1D), whereas the resistance from the pulmonary bed can be lumped into a 0D model. For example, with a model that has a 3D simulation for the aortic arch and the shunt, 0D models for pulmonary, coronary, upper and lower body circulations imposed from the inlet outlet or inlet boundaries at the pulmonary or systemic circulation bed (22, 23).

While insights could be gained from the hemodynamics in PDA and its impacts on the pulmonary and systemic circulations from the above mentioned models, the closure of PDA is an event associated with the remodeling of the arterial wall of the DA, which none of the hemodynamic models address directly. To that end a different category of models, i.e., for wall remodeling, is required.

## Multiscale Modeling for the Vascular Evolution

The DA in term infants is closed within a few hours after birth, because the increased arterial PaO2 and decreased circulating prostaglandin allow the smooth muscle media of the ductus to constrict (8). The constriction leads to ischemic hypoxia in the inner muscle wall of the DA, which consequently develops vascular endothelial growth factor that transform the ductus into a non-contractile ligament (8). In preterm infants, the intrinsic tone of the ductus is lower than that in full-term infants and has less capacity to constrict (1). Low fetal systemic arterial oxygen tension and circulating prostaglandin keep the lumen of DA patent.

Mechano-transduction plays an important role in the closure of DA: the wall shear stress acts on endothelial cells (ECs), triggering a cascade of intracellular events (24, 25). In addition, shear stress induced by transmural interstitial flow acts on the arterial media layer (Figure 1C) and induces prostaglandin production (26). In the *in vitro* smooth muscle cell gel model of (26), the production of prostaglandin E1 was enhanced with a 1 dyne/cm^2^ shear stress, but not by a 0.15 dyne/cm^2^ shear stress at any time.

The modeling framework for arterial remodeling has been proposed in several works (27, 28). For example, in such a model for aneurysms where the arterial wall remodels to accommodate complex arterial flows, the medial wall degradation is driven by an inflammatory response, followed by the changes in the distribution of collagen fibers. From mathematical modeling's perspective, the fibroblast-mediated collagen growth is governed by a system of ordinary differential equations to regulate the Transforming Growth Factor (TGF)-β, a key promoter of matrix deposition (28). However, efforts are required to assess whether these models are applicable to PDA and its closure.

## Discussion and Conclusion

The unique anatomical structure of the DA is a “double-edged-sword”. While it results in cardiac decompensation and pulmonary distress syndrome, it may also provide the only life-sustaining conduit to preserve pulmonary or systemic blood flow (3). While many preterm infants receive surgical procedures or pharmaceutical treatments for closing PDAs, it is still under debate whether a more invasive approach (e.g., surgeries) should be adopted, or a more conservative approach (e.g., machine taking) be adopted PDA be left alone.

Given the challenges in designing clinical or experimental protocols, *in silico* models are of great value to the studies of many subtle phenomena of PDAs. For example, the ductus in preterm infants is more sensitive to the vasodilating effects of nitric oxide and prostaglandin than full-term infants (1). *In silico* models could therefore be developed to check whether the arterial constituents and blood flow contribute to this phenomenon. This requires a multiscale approach where several *in silico* models are linked: the flow in the PDA is solved through a 0D-1D model, whereby the results are supplied to an arterial wall model of PDA for its modeling. The former model occurs at the vessel (millimeter) level, while the latter model occurs at cellular (e.g., collagen, micrometer) level.

An in-depth understanding of the underlying vascular physiology will also aid drug development for PDA. One example is that the drug for PDA, indomethacin, reduces renal, mesenteric and cerebral blood flows due to its role as the inhibitor of vasodilators, e.g., prostaglandin. Renal and cerebral flows occur in organ-level scales, while drug molecules access celluar membrane receptors at nanometer scales (3). A flow-limited pharmacokinetics model requires the data of blood flow rate to induce the amount of drug distributed to the organ (Figure 1D). Therefore, thoughts could be placed on coupling the blood flow arriving at the PDA with the pharmacodynamics effect of the drug, and the remodeling of the DA wall that spans tissue, cell and molecule levels (29). The circulation of prostaglandin and its signaling for the smooth muscle of the PDA provides an excellent example for such a multiscale model. These events are at the very core of the pathophysiology and clinical treatment of PDAs, where the application of *in silico* models is yet to be tested.

Multiscale and multi-modality modeling is a promising research direction in PDA and other arterial diseases in neonates and infants. With such a strategy, biomechanical simulations are connected with imaging and histological studies of the arterial wall. A class of mechano-biological models for growth and remodeling of the arterial wall and their intimate interaction with hemodynamics, cell activity, and arterial wall mechanics are described in (30). Specifically, in the work of (20), the three-dimensional flow simulations for a subject-specific carotid artery are compared with the endarterectomy specimen's histological maps, which could provide clues for cellular level models linking to the wall shear stress (WSS) that triggers arterial plaque. A roadmap for addressing the technical challenges for incorporating the proposed growth and remodeling into existing hemodynamic simulators are discussed in (29, 30).

In conclusion, we have provided a light literature review of the pathophysiology of PDA, and some *in silico* models used for blood flow and arterial wall modeling. We stress that a multiscale paradigm needs to be adopted when developing future *in silico* models for PDA and its treatment.

## Author Contributions

HH drafted the paper. XJ provided the medical context. HH and XJ reviewed and agreed the article. Both authors contributed to the article and approved the submitted version.

## Conflict of Interest

The authors declare that the research was conducted in the absence of any commercial or financial relationships that could be construed as a potential conflict of interest.

## Publisher's Note

All claims expressed in this article are solely those of the authors and do not necessarily represent those of their affiliated organizations, or those of the publisher, the editors and the reviewers. Any product that may be evaluated in this article, or claim that may be made by its manufacturer, is not guaranteed or endorsed by the publisher.
